# Spinal energy balance can predict post-operative spine alignment in Lenke 1 Adolescent Idiopathic Scoliosis

**DOI:** 10.1007/s43390-026-01353-7

**Published:** 2026-05-06

**Authors:** Tristan Langlais, Jérôme Sales de Gauzy, Joe Rassi, Mathilde Bony, Baptiste Brun-Cottan, Amandine Eon, Franck Accadbled, Pascal Swider, Pauline Assemat

**Affiliations:** 1 Institut de Mécanique des Fluides (IMFT), INPT, CNRS & Université de Toulouse, Toulouse, France; 2 Département de chirurgie orthopédique, traumatologique et réparatrice pédiatrique, Hôpital des Enfants, Centre Hospitalier Universitaire de Toulouse, Université de Toulouse, Toulouse, France

**Keywords:** Idiopathic scoliosis, Surgical planning, Biplanar radiograph, Energy approach

## Abstract

**Purpose:**

The preoperative planning of adolescent idiopathic scoliosis (AIS) remains largely debated. We hypothesized that adopting a biomechanical energetic framework could provide valuable insights for exploring the impact of spinal arthrodesis. Using this approach, we conducted a comparative analysis to quantify discrepancies between *in silico* simulations derived from preoperative radiographs and the actual three-dimensional spinal alignment obtained from post-operative imaging.

**Methods:**

Fifty-two consecutive patients with Lenke Type 1 AIS (mean age: 16 years; mean thoracic Cobb angle: 52°) who underwent posterior spinal fusion were included in the analysis. All patients had complete biplanar radiographs at three time points: preoperatively, post-operatively and at two-year follow-up. Discrepancies between *in silico* simulated surgery, calculated using preoperative radiographs and a biomechanical model, and actual clinical outcomes were quantified using two metrics: maximum coronal/sagittal deviations (MaxC/MaxS) from T1 to L5, and a comprehensive predictability factor (${\mathrm{a}}_{\mathrm{c}}$ and ${\mathrm{a}}_{\mathrm{s}}$) measuring cumulative 3D position discrepancies across 17 vertebral levels, normalized by total spinal length.

**Results:**

Mean MaxC was 4.7 mm (SD=4.9) and MaxS was 5.7 mm (SD=3.8). Mean values of ${\mathbf{a}}_{\mathrm{c}}$ were 3.4% (SD=3.8) and ${\mathbf{a}}_{\mathrm{s}}$ were 4.1% (SD=2.6). Of the cohort, 44 patients (90%) showed very good or good agreement in the coronal *in silico* simulation and 43 patients (88%) in the sagittal *in silico* simulation. When the coronal and sagittal results were combined, 38 patients (78%) showed very good or good agreement.

**Conclusion:**

The distribution of biomechanical energy obtained from preoperative radiographs is reliable to simulate spine alignment after arthrodesis in a Lenke 1 AIS cohort.

**Supplementary Information:**

The online version contains supplementary material available at https://doi.org/10.1007/s43390-026-01353-7.

## Introduction

Adolescent Idiopathic Scoliosis (AIS) leads to surgical treatment in 25% of cases when conservative orthopedic treatment proves ineffective [1]. Surgical treatment by arthrodesis aims to halt the progression of pathological curvature, to correct the three-dimensional spinal deformity and to achieve reliable long-term spinal fusion. Three-dimensional correction strives to address coronal alignment described by shoulders, waist and pelvis location and orientation. Restoration of sagittal balance between thoracic and lumbar curvatures, and reduction of axial vertebral rotation in the horizontal plane to minimize rib prominence, are also targeted. Patient comfort and preservation of spinal mobility are the main parameters for preoperative planning of location and extent of arthrodesis. Inadequate correction may lead to malalignment [2] provoking significant post-operative complications. Major reported complications [3] are adding-on of non-instrumented curve and proximal or distal junctional kyphosis which often lead to implant breakage or pull-out. Beyond the choice of instrumented levels, complications are strongly impacted by the amount of corrections applied [4, 5]. It appears that preoperative planning still remains a matter of debate, and that the strategy for combining patient comfort with complications minimization remains an open question.

To study the complex behavior of AIS, a significant number of methodologies have been implemented. They have generally been developed using engineering tools dedicated to *in silico* modeling. Literature review reports some semi-analytical or multi-body discrete models [6–10] but most of them are using the finite element method to study etiopathogenesis [11, 12] or prediction of clinical outcomes [13]. The ability of the finite element method to capture clinical reality shows limitations because of the non-standardization of clinical setting with mandatory patient-dependence of surgical strategy. Anatomy may be partially accessible through clinical imaging on a macroscopic scale, but pathological tissue properties, boundary conditions, i.e., kinematic and loading conditions and their temporal evolutions are generally inaccessible in clinical setting. In addition, pathological events and treatment impacts are involved in a reactive loop that is difficult to grasp and predict. To our knowledge, no strategy has yet been proposed for predicting AIS surgical outcomes, taking into account the unavoidable limitations of clinical reality.

**Fig. 1:**
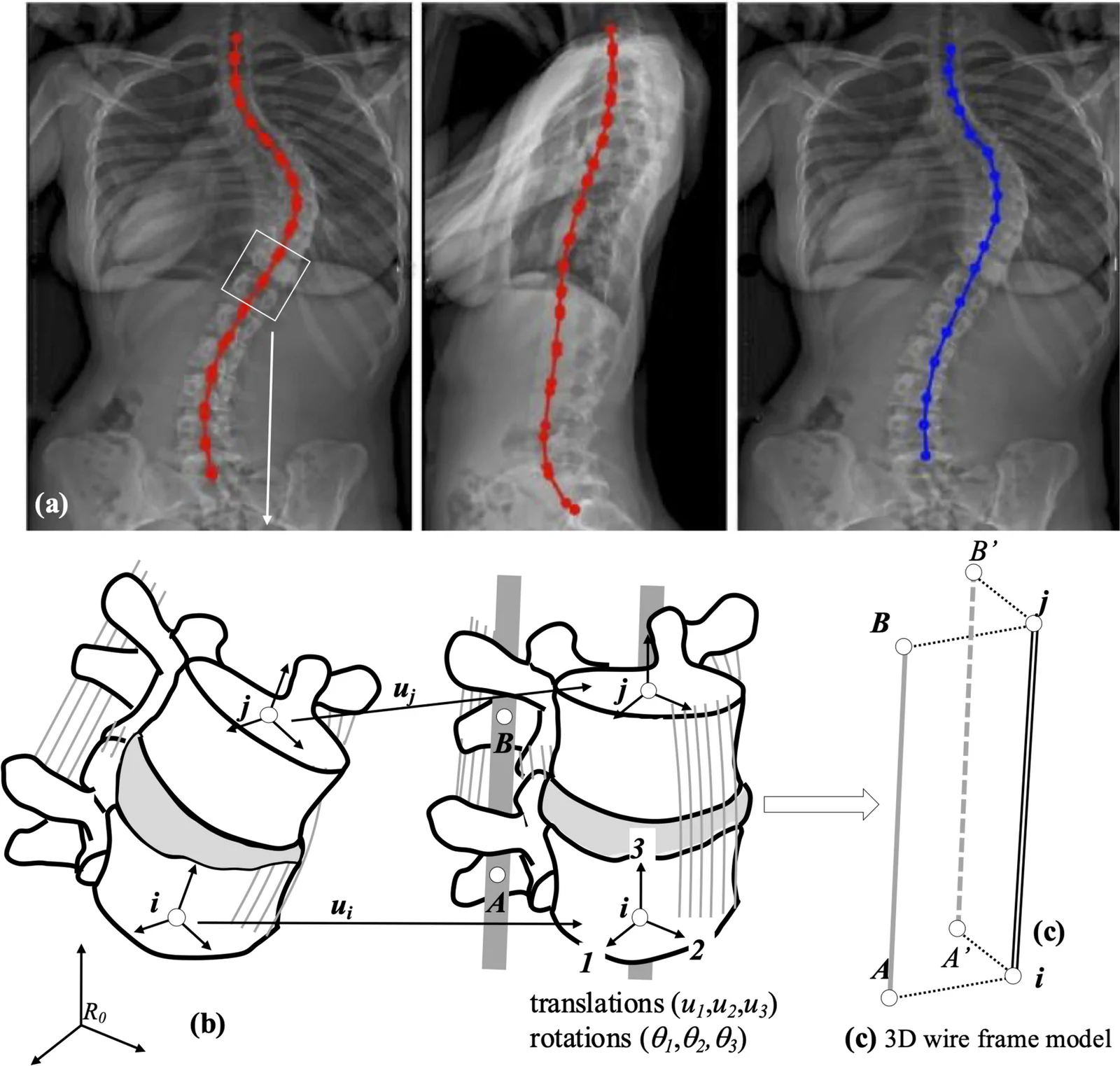
From clinical imaging to energy-based 3D wire frame model of scoliotic spine. **a** X-ray imaging (EOS®) showing spine deformation in the frontal and sagittal planes; Illustration of the 3D wireframe geometry obtained by segmentation in image J. Centers of end plate are segmented in the coronal and sagittal planes in red, spinous processes are segmented in blue to reconstruct wire structure in the transverse plane. **b** vertebral body kinematics *u*_*i*_ and ***u***_*j*_ describing spine correction in the presence of arthrodesis rods. **(c)** Updated 3D wire frame model based upon energy minimization and involving surgical instrumentation

To overcome these limitations, we proposed a holistic approach whose central principle considers the spine as a quasi-conservative thermodynamic system at the macroscopic scale with stationary equilibria associated with total energy minima [14]. Our top-down analysis considers succession of vertebral segments, as schematically depicted in Figure 1. In thermodynamic terms, internal energy accounts for strain energy of deformable structures, i.e., discs, ligaments, facet joints, and vertebral endplates, while external work accounts for the work of intersegmental ligaments, muscle actions, and gravity. Both internal and external forces derive from local energy potentials in the vicinity of successive quasi-static equilibria, and this results in global non-linear response segmented into a piecewise linear model at each step where vertebral bodies kinematics and effective biophysical tensors are describing the macroscopic mechanical response of the segments. Kinematics and biophysics were identified using an inverse algorithm nourished by imaging, i.e., biplanar X-rays obtained in clinical routine. The methodology gives access to the complete energy balance within the spine. Results showed that biophysical energy provided a relevant framework for characterizing spinal alignment in AIS patients [14].

The community is still debating a robust methodology for surgical planning. We hypothesized that the overall post-operative alignment of the spine with arthrodesis could be explored using the redistribution patterns of biophysical energies and predicted using the patient's radiographic preoperative record. We conducted a comparative analysis, measuring discrepancies between *in silico* simulations and the actual three-dimensional spinal alignment reconstructed from post-operative imaging.

## Materials and methods

### Study design and patients’ selection

This study was registered in the National Commission on Informatics and Freedoms (CNIL) database register (No 2239822). All participants provided informed consent prior to data collection and analysis, in accordance with institutional ethical standards. We conducted a retrospective analysis of a consecutive patient cohort treated between September 2017 and August 2020. Inclusion criteria were AIS classified as Lenke 1 [15], with a right thoracic curvature, undergoing posterior vertebral arthrodesis, with no prior surgical treatment or halo gravity preparation, and with a complete follow-up. Complete follow-up was defined as comprehensive clinical assessment and radiographic evaluation at three time points: preoperatively, three months post-operatively, and at final follow-up (with a minimum of two years). All imaging studies were digitally archived within the hospital Picture Archiving and Communication System (PACS). All included patients underwent biplanar low-dose coronal and sagittal radiographs (EOS^®^, EOS imaging, Paris, France) in the standard position [16] one month before surgery, at three months after surgery and at final follow-up.

**Fig. 2:**
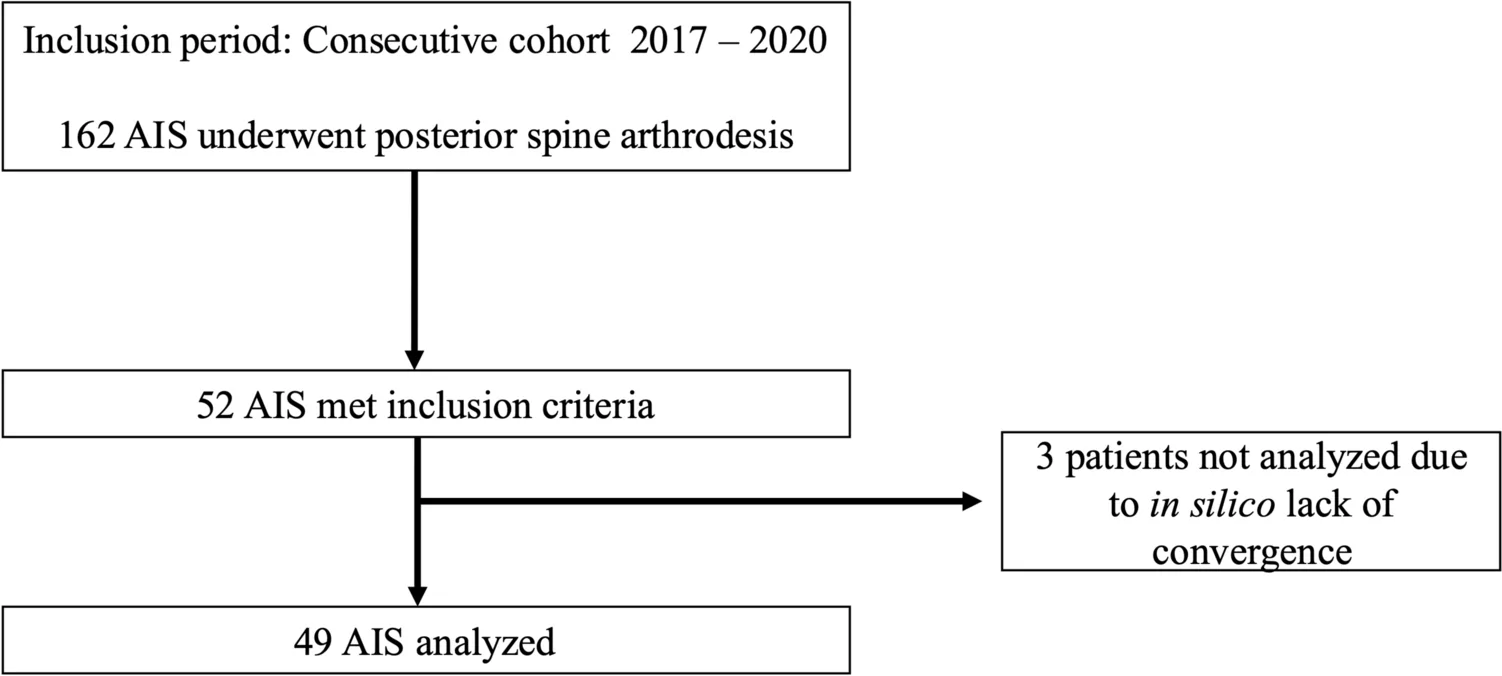
Flow chart

One hundred and sixty-two AIS patients underwent posterior fusion correction consecutively during the inclusion period, and 52 patients met the inclusion criteria (flow chart in Figure 2). All posterior correction fusions were performed by a single surgeon using a standardized technique [17]. A posterior translation correction with concave thoracic sublaminar bands was performed. The upper end of the construct was fixed with a clamp [18] (transverse hook and multiaxial pedicle screw), and the lower end with multiaxial pedicle screws. Instrumentation consisted of a chrome-cobalt alloy rod of 5.5 mm diameter (Solera, Medtronic^®^, Dublin, Ireland).

Demographic, clinical and radiographic characteristics are summarized in Table 1. Among the 52 patients, four patients (7.7 %) required secondary surgical intervention during the follow-up period. The indications for reoperation were diverse: two patients underwent transverse connection removal due to persistent thoracic discomfort; one patient developed a surgical site infection at three weeks post-operatively, necessitating surgical drainage and a patient experienced bilateral rod fracture in the lumbar region at four years after the arthrodesis, requiring revision surgery with domino connection. The mean follow-up duration for the entire cohort was 3.0 years (range: 2.0-6.0 years).

**Table 1:** Cohort characteristics

	Cohort: *n* = 52 AIS included
Age of surgery (years)	16 (3); 12–26
Body mass index (kg/cm^2^)	21 (3); 14–28
Lenke classification (*n*)	
Lumbar modifier	A: *n* = 34; B: *n* = 11, C: *n *= 7
Thoracic sagittal profil modifier	*N*: *n*=41; - : *n* = 11
Upper instrumented vertebrae (*n*)	T2: *n *= 2; T3: *n *= 30; T4: *n *= 17; T5: *n *= 3
Lower instrumented vertebrae (*n*)	L1: *n *= 16; L2: *n *= 15; L3: *n *= 16; L4: *n *= 5
Number of instrumented levels (*n*)	11 (1); 9–14
Number of concave thoracic sublaminar bands (*n*)	3 (0.5); 3–5
Preoperative thoracic cobb angle (°)	52 (9); 33–78
3 months post-operative thoracic Cobb angle (°)	12 (5); 1–26
Last follow-up post-operative thoracic Cobb angle (°)	12 (5); 1–26
Preoperative T5-T12 sagittal angle (°)	16 (10); 2–43
3 months post-operative sagittal angle (°)	16 (9); 2–51
Last follow-up post-operative sagittal angle (°)	17 (7); 4–40
Re-operations	*n *= 4
Deep infection	*n *= 1
Rod breakage	*n *= 1
Transverse connection removal	*n* = 2
Follow-up (years)	3 (1); 2–6

n represents the number Results are presented as mean (Standard deviation); range

### Energy-based method of surgical modeling and outcome assessment

**Fig. 3:**
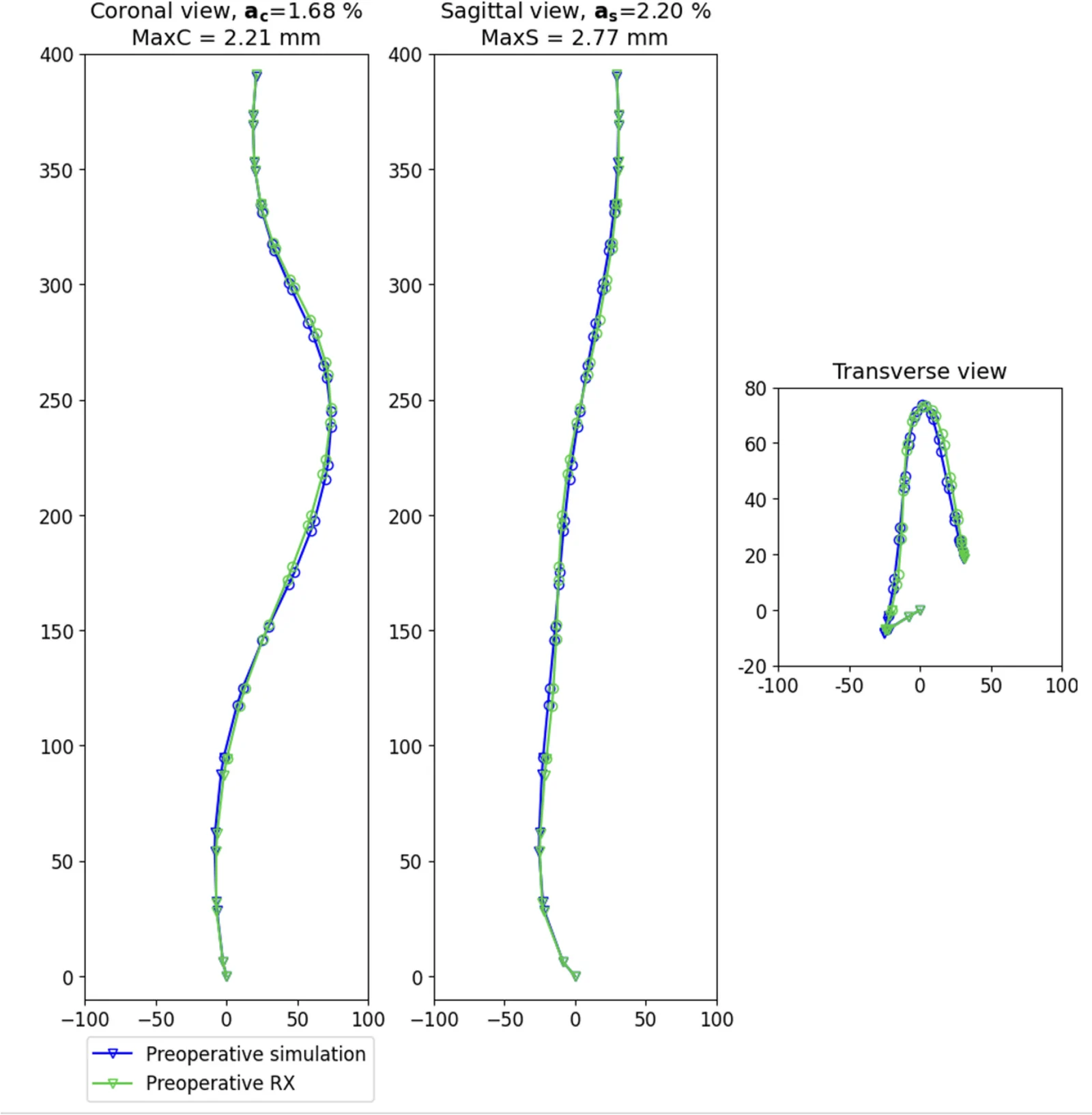
A comparison between numerical simulation of the preoperative case and the clinical RX using a wireframe representation in the 3 planes of space for one patient. Green curves represent the real geometry and blue curves represent the simulation. Axes are expressed in mm. MaxC corresponds to the maximum discrepancies in the coronal plane, MaxS in the sagittal plane, **a**_**c**_ is the predictability factor in the coronal plane, **a**_**s**_ in the sagittal plane

The first step was to segment spinal components to implement the preoperative 3D wire frame model (Figure 1a), which includes the center of vertebral endplate, i.e., points *i* and *j* in Figure 1b and centers of the spinous process from T1 to L5 in the coronal and sagittal X-ray images (Image J^®^). Second, the energy method supported by the inverse algorithm was used to identify effective tensors describing stiffnesses, and the work of muscles, ligaments, and gravity at equilibrium [19] (SPINERGY software^®^) and to derive energy balance in frontal, sagittal and horizontal planes using preoperative X-rays [14], see Figure 3. Details are given in appendix A1. Numerical convergence is reached when a minimum is found by the algorithms for the inverse problem [14]. This convergence ability is related to the mathematical properties of the energy function for each patient.

The third step concerned the *in silico* surgery simulation (see appendix A2) and was based on the knowledge of the instrumentation levels and their locations, the instrumentation sagittal shape, as well as the reduction at the upper and lower instrumented levels. Instrumentation modified effective tensors in the arthrodesis zone with significant additional stiffness depending upon rod geometrical properties, material, and span and which were straightforwardly obtained from the exact solution of slender beam quasi-static responses [20, 21]. As shown in Figure 1c, rods, i.e., points A and *B*, were rigid body attached to vertebral bodies *i* and *j*. The size and location of arthrodesis were determined based on the patient's surgical plan. Additional stiffness K_r_*,* due to the rod, was described analytically and combined with the initial stiffness tensors K of the segment and the methodology was described by the set of equations in appendix A2 (equations 3, 4 and 5). The contribution in extension, shear, flexion, and torsion depending on diameter, span, offset and material properties of rods, were detailed in expression (6).

Clinical outcome was established using 3 months post-operative X-rays biplanar records. First, the *in silico* prediction of Cobb angles and counter-curvature angles in the coronal plane were examined. Next, a local evaluation of discrepancies between *in silico* simulation and clinical outcome was based upon two criteria in coronal and sagittal planes. The first criterion was local and defined as greater the distances MaxC and MaxS, in coronal and sagittal planes, respectively, between clinical image and *in silico* simulation. The second criterion called the predictability factor **a** (%), was global while considering the seventeen vertebral levels, and was defined as the distances sum over *m* vertebral bodies scaled by the curvilinear length *l* as follows:

**a** (%) = (1/*l*) ∑_*m*_ (*x*_*m*_^*2*^_*model*_-* x*_*m*_^*2*^_*clinic*_)^0.5^ where* x*_*m*_ gives the three-dimensional coordinate of vertebral bodies from T1 to L5. The percentage ${\mathbf{a}}_{\mathrm{c}}$ and ${\mathbf{a}}_{\mathrm{s}}$ were values of this factor in coronal and sagittal planes, respectively**.**

Finally, agreement for both coronal and sagittal planes, was arbitrarily stratified as follows, based on clinical practice:

- very good agreement with ${\mathbf{a}}_{\mathrm{c}}$ or ${\mathbf{a}}_{\mathrm{s}}$ lower than 5 % and MaxC or MaxS lower than 5 mm
- good agreement with ${\mathbf{a}}_{\mathrm{c}}$ or ${\mathbf{a}}_{\mathrm{s}}$ lower than 10 % and MaxC or MaxS comprises between 5 and 10 mm or ${\mathbf{a}}_{\mathrm{c}}$ or ${\mathbf{a}}_{\mathrm{s}}$ comprises between 5 and 10 % and MaxC, MaxS lower than 10 mm
- moderate agreement with ${\mathbf{a}}_{\mathrm{c}}$ or ${\mathbf{a}}_{\mathrm{s}}$ lower than 15 % and MaxC or MaxS comprises between 10 and 15 mm or ${\mathbf{a}}_{\mathrm{c}}$ or ${\mathbf{a}}_{\mathrm{s}}$ comprises between 10 and 15 % and MaxC or MaxS lower than 15 mm
- poor agreement with ${\mathbf{a}}_{\mathrm{c}}$ or ${\mathbf{a}}_{\mathrm{s}}$ greater than 15 % and MaxC or MaxS greater than 15 mm

To summarize, *in silico* output includes MaxC, ${\mathbf{a}}_{\mathrm{c}}$, the Cobb and counter-curvature angles as well as energy and stiffness over 17 vertebral segments in the coronal plane. In the sagittal plane, MaxS, ${\mathbf{a}}_{\mathrm{s}}$, energy and stiffness over 17 vertebral segments are explored whereas in the transversal or horizontal plane torsion and transversal energy are illustrated.

### Statistics

Data normality and heteroskedasticity were assessed with the Shapiro–Wilk test and the Levene’s test. Continuous outcomes were compared with the Kruskall–Wallis test, Mann–Whitney test, ANOVA test or Wilcoxon sign-rank test according to data distribution. Non-continuous outcomes were compared with Fisher’s exact test according to data distribution. If the null hypothesis of the test was rejected, post hoc pairwise analyses were performed with Tukey’s HSD test. Alpha risk was set to 5% (*α* = 0.05). The results of the statistical tests are presented in Tables 2 to Table 3, Table 4 and 5. Statistical analysis was performed using EasyMedStat 3.42 (EasyMedStat^®^).

**Table 2:** Outcome assessment according Lenke’s classification modifiers

	${\mathbf{a}}_{\mathrm{c}}$(%)	MaxC (mm)	${\mathbf{a}}_{\mathrm{s}}$(%)	MaxS (mm)
Lumbar spine modifier				
A	2.1 (IQR 1.9)	3.1 (IQR 2.7)	2.9 (IQR 6.4)	4.5 (IQR 4.5)
B	2.6 (IQR 1.8)	4.1 (IQR 3.7)	3.3 (IQR 3.1)	3.8 (IQR 0.9)
C	2.8 (IQR 11.4)	3.9 (IQR 1.2)	3 (IQR 1.1)	4.5 (IQR1.9)
*p-values*	*0.4*	*0.4*	*0.6*	*0.7*
Thoracic sagittal profile modifier				
Hypo	1.7 (IQR 2.5)	2.3 (IQR 2.6)	2.6 (IQR 1.4)	3.8 (IQR 1.7)
Normal	2.5 (IQR 1.8)	3.4 (IQR 3.2)	3.5 (IQR 3.5)	4.7 (IQR 5)
*p-values*	*0.6*	*0.5*	*0.07*	*0.1*

Results are presented as mean (IQR = Interquartile Range)

**Table 3:** Degree of agreement between simulations and X-ray data 3 months post-operative

	Coronal (*n* = 49)	Sagittal (*n* = 49)	Combination of coronal and sagittal (*n* = 49)
Very good agreement	36 (74%)	30 (61%)	24 (49%)
Good agreement	8 (16%)	13 (27%)	14 (29%)
Moderate agreement	2 (4%)	4 (8%)	6 (12 %)
Poor agreement	3 (6%)	2 (4%)	5 (10%)

Values represent the number of patients in each group accompanied with the percentage in the *n* = 49 cohort

**Table 4:** Demographic, surgery and radiographic parameters according to the coronal agreement

	Very Good *n* = 36	Good *n* = 8	Moderate and poor *n* = 5	*p-value*
Body mass index (kg/cm^2^)	20.6 (SD 3)	20.3 (SD 2.8)	21.9 (SD 3.4)	*0.6*
Lumbar spine modifier : n (%)				
A	25 (69.4%)	6 (75%)	1 (20%)	*0.1*
B	7 (19.4%)	2 (25%)	2 (40%)	
C	4 (11.1%)	0	2 (40%)	
Thoracic sagittal profile modifier : n (%)				
Hypo	7 (19.4%)	1 (12.5%)	1 (20%)	*0.9*
Normal	29 (80.5%)	7 (87.5%)	4 (80%)	
Preoperative thoracic cobb angle (°)	50.5 (SD 8.3)	54.5 (SD 13)	49.8 (SD 10.4)	*0.6*
Preoperative T5-T12 sagittal angle (°)	15.4 (SD 10.3)	23.4 (SD 7)	16.6 (SD 7)	*0.08*
Number of instrumented levels	11.3 (SD 1.4)	10 (SD 0.9)	10.4 (SD 0.9)	*0.07*
Correction of coronal thoracic cobb angle (%)	76%	76%	72%	*0.6*

*n* represented the numbers of patient

**Table 5:** Demographic, surgery and radiographic parameters according to the sagittal agreement

	Very Good *n* = 30	Good *n* = 13	Moderate and poor *n* = 6	*p-value*
Body mass index (kg/cm^2^)	20.9 (SD 3.3)	20.4 (SD 2.6)	20.5 (SD 1.4)	*0.9*
Lumbar spine modifier : *n* (%)				
A	20 (66.7%)	10 (76.9%)	2 (33.3%)	*0.1*
B	6 (20%)	1 (7.7%)	4 (66.7%)	
C	4 (13.3%)	2 (15.4%)	0	
Thoracic sagittal profile modifier : *n* (%)				
Hypo	21 (77.8%)	12 (80%)	4 (80%)	*0.9*
Normal	6 (22%)	3 (20%)	1 (20%)	
Preoperative thoracic Cobb angle (°)	50.5 (SD 9.3)	53.6 (SD 10)	48.5 (7.4)	*0.4*
Preoperative T5-T12 sagittal angle (°)	13.3 (SD 8.7)	22.1 (SD 10.2)	22.8 (SD 7.5)	*0.006*
Number of instrumented levels	11.3 (SD 8.7)	10.7 (SD 1)	10 (SD 0.6)	*0.06*
Correction of T5-T12 Sagittal angle (%)	50%	26%	25%	*0.07*

*n* represented the numbers of patient

## Results

Among 52 patient records examined, only three (6%) did not show numerical convergence.

First, preoperative segment rotational stiffness was examined due to its clinical relevance. This gave effective average values in the order of 1.1 × 10^3^ N.m/rad in frontal flexion, 1.5 × 10^4^ N.m/rad in sagittal flexion, and 2 × 10^4^ N.m/rad in torsion in the horizontal plane. These magnitudes were consistent with the literature, which did, however, report significant variations [8, 14, 22]. The stiffness contribution of arthrodesis on the free levels was significant, with an addition of around 30×10^4^ Nm/rad in the coronal and sagittal flexion planes and 1.1×10^4^ Nm/rad in torsion, for the most clinically relevant and energetic terms, i.e., diagonal terms associated with rotations as detailed in appendix A2 (equation 6).

Secondly, the median difference between post-operative and predicted Cobb angles was 0.93° (SD= 1.73°, CI 95% = 0.8-1.78) with *p* < 0.001. The median difference between post-operative and predicted counter-curvature angles was − 1.51° (SD= 5.23° , CI 95% = − 3.42 - − 0.87) with *p* < 0.001. These results show a very good prediction of Cobb and counter-curvature angles by the *in silico* approach.

Detailing the results at the vertebral segment scale, among the 49 converged calculations, mean values of MaxC were 4.7 mm (SD=4.9 mm; CI 95% = 3.3-6.1 mm) and MaxS 5.7 mm (SD=3.8 mm; CI 95% = 4.6-6.8 mm) in the coronal plane and the sagittal plane, respectively. Mean values of ${\mathbf{a}}_{\mathrm{c}}$ were 3.4 % (SD=3.8%; CI 95% = 2.3-4.5%) and ${\mathbf{a}}_{\mathrm{s}}$ were 4.1 % (SD=2.6%; CI 95% = 3.3–4.8%) in the coronal plane and in the sagittal plane, respectively. There was no difference in assessed outcomes according to the lumbar and sagittal modifiers of the Lenke classification as shown in Table 2.

**Fig. 4:**
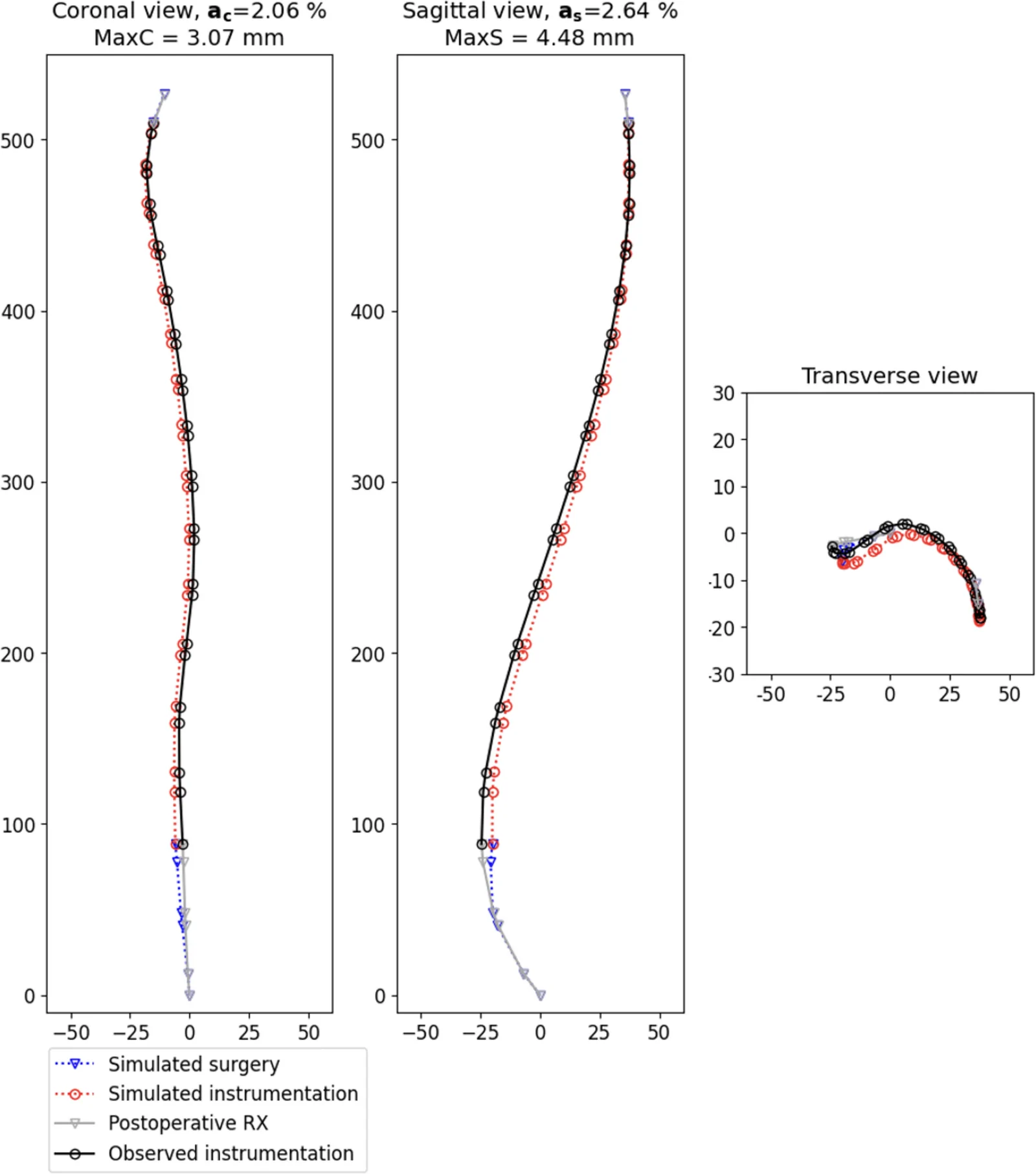
A comparison between numerical simulation of surgery and post-operative surgery using a wireframe representation in the 3 planes of space. Black and gray curves represent the real geometry whereas blue and red represent the simulation. Gray and red correspond to the instrumented levels whereas black and blue the free vertebrae segments. Axes are expressed in mm. MaxC corresponds to the maximum discrepancies in the coronal plane, MaxS in the sagittal plane, **a**_**c**_ is the predictability factor in the coronal plane, **a**_**s**_ in the sagittal plane. Example of a patient with very good agreement. Examples for good agreement, moderate agreement and poor agreement can be found in supplementary material

The relevance of the energy model to predict clinical outcome is summarized in Table 3, where the patient cohort was stratified into four groups. Of the cohort of 49 patients on which surgery calculations could be performed, 44 patients (90%) showed very good or good agreement in the coronal *in silico* simulation. Similarly, 43 patients (88%) showed very good or good agreement in the sagittal *in silico* simulation. When the coronal and sagittal results were combined, 24 patients (49%) showed very good agreement, 14 patients (29%) showed good agreement, 6 patients (12%) showed moderate agreement, and 5 patients (10%) showed poor agreement. Figure 4 depicts the patterns associated with the very good agreement group and other groups illustrations can be found in supplementary materials.

In addition, *in silico* results were examined according to anatomical planes and summarized in Table 4 and 5. For coronal *in silico* simulation, there was no difference in agreement according to demographic, surgical, or radiographic parameters Table 4 whereas in Table 5 for sagittal *in silico* simulation, mean preoperative T5-T12 kyphosis was 13.3 (SD 8.7) in the very good agreement group (*n*=30) and 22.1 (SD 10.2) in the good agreement group (*n*=13). Pairwise analyses revealed differences between these groups (*p*=0.015).

**Fig. 5:**
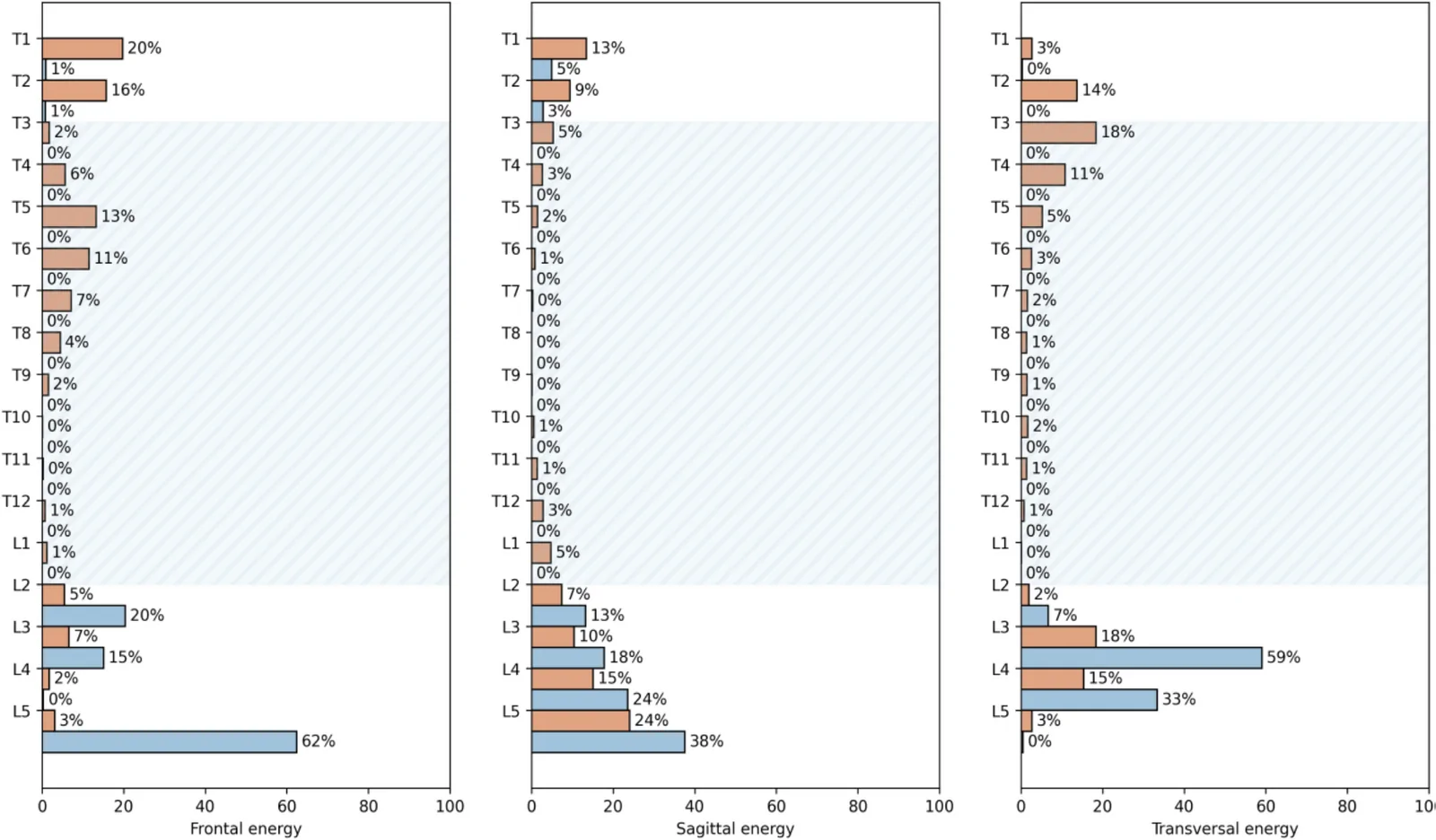
Prediction of strain energy redistribution in an operated spine with T3-L2 arthrodesis. Orange plots concerned the preoperative energy balance, while blue plots corresponded to the post-operative energy balance. Segment energies were scaled to the spine overall energy and expressed as percentages

Finally, strain energy distribution profiles, namely *U*_*i*_ from equation (1, 2) for segments *i*=1…17, were shown in Figure 5. The selected illustration was representative of the cohort since the Cobb angle was 50° with a lumbar spine modifier and T3-L2 arthrodesis. Segment energies were scaled according to the spine overall energy, and a drastic reduction in strain energy was observed in the arthrodesis zone. Energy was redistributed in segments remaining free especially below the arthrodesis. A significant increase was found with mean values of +20% SD27, +10% SD 4 and +15% SD 19 in frontal, sagittal and horizontal planes, respectively. When the three planes were combined, the overall increase was +35% SD 20.

## Discussion

We first hypothesized that focusing on the energetic approach might be relevant for exploring and interpreting the impact of arthrodesis in operated patients and, beyond that, might provide indications for surgical planning. We carried out a clinical study on a cohort of 52 patients, and the results showed that in 80% of cases, our hypothesis was validated, since we obtained very good to good agreement between the predictive *in silico* simulation and the clinical results.

Numerous finite element models of AIS with increasing complexity have been proposed to corrected pathological deformations [2, 6, 7, 9, 13, 23, 24] [25] while generally focusing on the post-operative coronal Cobb angle. This finite element approach is the main method found in the literature to investigate the complex mechanical processes underlying surgical scoliosis correction and post-operative spinal adaptation. Limitations of such methods may include high computational cost due to the size of the problems for whole musculoskeletal systems, which can involve non-linear responses, inter-patient variations in material properties, particularly for pathological tissues, and difficulties in identifying boundary conditions and kinematic loads *in vivo* [26]. More recent approaches explored artificial intelligence capabilities software used for surgery alignment predictions limited to 75 % accuracy [27] with significant limitation of biophysical and clinical non-explicability. Regression techniques are also explored in the literature with limited patient-specific prediction possibilities [28].

To overcome these obstacles, we proposed obtaining spine balance by minimizing the total energy function. Our methodology was based on the fundamental principle of work minimization to determine an equilibrium position, namely Hamilton’s principle [34]. The finite element method, commonly used in engineering and biomechanics, applies this principle, followed by the weak formulation of virtual work combined with a multiscale description. The methodology can be described as holistic due to its comprehensive nature, with the smallest scale of analysis being that of the vertebral segment in its entirety described by effective biophysical properties. It was specific to each patient while relying on routine biplanar X-rays, with ergonomics and computational costs compatible with clinical practice. Depending on computing capabilities, the time required on a personal computer for the 3D preoperative digital wireframe model to converge was around one hour, while surgical simulation took only ten to thirty seconds. These times were compatible with the clinical context and constraints. In addition, the methodology assesses in near-real time the impact of instrumentation modification in terms of location, extent and shape on patient alignment. The predictive surgery *in silico* simulation showed discrepancies in combined coronal and sagittal distances of up to 10% between predicted spine position and shape and actual post-operative outcomes, with maximum distances less than 10 mm in 80% of patients. Curvature corrections were also predicted with very good accuracy, as deviation was less than 1.5 degrees. These results were considered very good given the cumulative uncertainties in patient management, imaging, and intraoperative adaptation associated with instrumentation and clinical procedures. Another interesting finding was that agreement outcomes were not impacted by the magnitude of the preoperative Cobb angle and the curvature types, i.e., lumbar and thoracic sagittal modifiers according to the Lenke classification. Regarding the difference between coronal and sagittal alignment, particularly as a result of the T5-T12 thoracic kyphosis, the study found that modeling was sensitive to the orientation of the spinous process of the sacrum relative to the horizontal plane. Uncertainties intrinsic to anatomy increased in this region.

Without any invasive procedures, as it relied solely on radiographs, energy analysis traced back to the internal strain energy. It was found that the local stiffness of the instrumentation led to a drastic reduction in the contribution of spinal fusion to the overall energy balance to the detriment of the non-operated segments. This redistribution toward remaining free segments exhibited a significant gradient at the instrumentation transition. This phenomenon, accurately predicted by the model, could precede reported complications, such as worsening of the non-instrumented curvature, proximal or distal junctional kyphosis, which often lead to implant pull-out or breakage, as well as premature aging of lumbar discs [3, 29–32].

Accuracy and reproducibility of image segmentation, which still depended in part on the surgeon, led to limitations. Indeed, as our protocol had to be adapted to clinical routine and its constraints, it was dependent on consecutive image series of potentially variable quality, thereby preceding discrepancies in key anatomical landmark detection. Modeling proved to be sensitive to the orientation of the sacrum spinous processes relative to the horizontal plane, and uncertainties increased in this region. This could contribute to differences between coronal and sagittal alignment predictions, particularly due to the T5-T12 thoracic kyphosis. It appears that *in silico* outcomes were influenced by the statistical impact of patients with flat backs, for whom sagittal reconstruction was more reproducible. This will need to be confirmed in future cohorts. Non-convergence was observed in three out of fifty-two cases, or less than 6 % of the cohort. Combining our software with guess generator algorithms for the inverse problem may improve the convergence rate on the cohort, and exploring larger cohorts will allow this constraint to be relaxed while verifying its eligibility for clinical outcomes in routine.

Another limitation is that transverse planes were indicated in the figures as illustrations of the spine pre- and post-operative 3D distribution but were not quantitatively detailed [33]. These will be further studied in future work, as will the comparison of our results with the two-year follow-up, which qualitatively does not appear to show significant variation in spine alignment compared to the post-operative three-month alignment.

Moreover, the clinical relevance of the energy method was evaluated in a Lenke 1 AIS cohort. Although this corresponded to a well-described and frequently encountered group, it could constitute a limitation. In any case, there should be no obstacle to its evaluation in larger differentiated cohorts.

Future work will focus on reducing the sensitivity of the energy model to clinical data dispersions, such as local spine–pelvis orientations, as well as improving computation times and ergonomics adapted to clinical users and contexts. In addition, the clinical relevance will benefit from feedback from multicenter studies involving different AIS cohorts and clinical practices. The advantage of this type of simulation is that it allows anticipation of the overall post-operative alignment, as well as the behavior of the curvatures above and below the instrumentation. Ultimately, this approach should help clinicians select the appropriate levels for instrumentation, and this decision can be further supported by longer-term follow-up of the clinical cohort.

## Conclusion

The distribution of biomechanical energy obtained from preoperative radiographs is reliable for simulating patient-specific spine alignment post-operatively in a Lenke 1 AIS cohort. In addition, exploration of energies could enable modeling of the curve variation above and below the instrumented area for different surgery planning.

## Electronic supplementary material

Below is the link to the electronic supplementary material.

Supplementary file1 (TIFF 252 KB)

Supplementary file2 (TIFF 253 KB)

Supplementary file3 (TIFF 240 KB)

## Data Availability

The datasets generated during and/or analyzed during the current study are available from the corresponding author on reasonable request.

## Appendix

### Energy-based method

According to Hamilton's principle [34], the holistic approach considers spinal balance to be associated with a minimum total energy *V* [14], with cancelation of the first derivative with respect to kinematics, as expressed by equation (1). Generic forms of strain energy *U*, muscles and ligaments works *B,* and gravity works *G,* were expressed by equation (2).

1 $$V(u,p)\; = \;U(u,p) + B(u,p) - G(u,p)\;with\;\partial V(u,p)/\partial u\; = \;0$$

2 $$\begin{array}{*{20}c} {U\; = \;\frac{1}{2}\mathop \sum \limits_{i} q_{i}^{t} K_{i} q_{i} } & {B\; = \;\frac{1}{2}\mathop \sum \limits_{i} q_{i}^{t} B_{i} q_{i} } & {G\; = \;} \\ \end{array} \mathop \sum \limits_{i} G_{i} q_{i}$$

As a corollary, forces deriving from local potentials enabled the non-linear response of the spine to be reconstructed using a succession of piecewise linear responses. This introduced quadratic and linear mathematical operators related to vertebral segments. Generalized coordinates *q*_*i*_ introduced in equation (2) and relative to vertebral segment *i*, involved kinematics, i.e., three translations and three rotations, and components of effective biophysical tensors such as the effective stiffness tensors K_i_ , whose components are included in the parameter vector *p*. In addition, the reference state was also taken into account in *q*_*i*_ to describe the initial energetic state which impacted the non-linear response. The reference position corresponded to an asymptomatic spine, i.e., with no pathological deformities in any of the three anatomical planes. Kinematics and biophysics were iteratively identified using an inverse mathematical algorithm nourished by imaging, i.e., biplanar X-rays obtained in clinical routine. Convergence of the iterative process was established by minimizing the distance in a mathematical sense, of pathologic and reference segment kinematics. The methodology was implemented in dedicated software designed for the clinical environment (Spinergy software ®) [19] and the full model is described in [14].

### Surgery modeling

The surgical rods modified the wire model of the spine and specifically the effective stiffness tensors *K*. Our modeling considered the rod as a cylindrical beam *A*-*B* clamped with an offset in the posterior zone of the vertebral body as described in Figure 1c. Continuum mechanics [20] provided the stiffness matrix *k*_*r*_ [12x12] from a kinematics of 3 translations (*u*_*1*_,*u*_*2*_,*u*_*3*_) and 3 rotations (θ_*1*_, θ_*2*_*,* θ_*3*_) expressed in the segment reference frame at points *A* and *B*, with symmetry conditions of submatrices such that *k*_*rAA*_ and *k*_*rAB*_ were equal to *k*_*rBB*_ and *k*_*rBA*_, respectively. Topologies and coefficients were expressed as follows:

3 $$k_{rAA} = \left[ {\begin{array}{*{20}c} {k_{11} } & 0 & 0 & 0 & 0 & 0 \\ {} & {k_{22} } & 0 & 0 & 0 & {k_{26} } \\ {} & {} & {k_{22} } & 0 & { - k_{26} } & 0 \\ {} & {} & {} & {k_{44} } & 0 & 0 \\ {} & {sym} & {} & {} & {k_{55} } & 0 \\ {} & {} & {} & {} & {} & {k_{55} } \\ \end{array} } \right];\, k_{rAB} = \left[ {\begin{array}{*{20}c} { - k_{11} } & 0 & 0 & 0 & 0 & 0 \\ 0 & { - k_{22} } & 0 & 0 & 0 & {k_{26} } \\ 0 & 0 & { - k_{22} } & 0 & { - k_{26} } & 0 \\ 0 & 0 & 0 & { - k_{44} } & 0 & 0 \\ 0 & 0 & {k_{26} } & 0 & {k_{511} } & 0 \\ 0 & { - k_{22} /2} & 0 & 0 & 0 & {k_{511} } \\ \end{array} } \right]$$

$$k_{11} \; = \;\frac{es}{L};\,k_{22} \; = \;\frac{12el}{{2l^{3} (1 + \lambda )}};\;k_{44} \; = \;\frac{gJ}{L};\,k_{55} \; = \;\frac{(4 + \lambda )eI}{{L(1 + \lambda )}};\,k_{26} \; = \;\frac{6eI}{{L^{2} (1 + \lambda )}};\,k_{511} \; = \;\frac{(2 - \lambda )eI}{{L^{2} (1 + \lambda )}}\,and\,\lambda \; = \;\frac{12eI}{{\alpha gsL^{2} }};s\; = \;\frac{{\pi \phi^{2} }}{4};\,I\; = \;\frac{{\pi \phi^{2} }}{64};J\; = \;\frac{{\pi \phi^{4} }}{32};g\; = \;\frac{e}{2(1 + \upsilon )}$$

Non-zero coefficients described stiffness in extension, flexion, shear, torsion, and associated couplings. Parameters *ϕ*, *L*, *s*, *I*, *J*, and *α* were diameter, length, cross-sectional area, bending quadratic moment, torsion quadratic moment, and shear coefficient. The material properties were Young modulus *e* and Poisson ratio *υ*.

The assembling of the rod stiffness matrix with the segment stiffness matrix calculated in A1 assumed a rigid body motion of the rod fixation *A* - *B* with vertebral bodies *i*–*j* which has been described by the geometric transformation *P*_*ij*_ [12×12] involving offsets *a* and *b*. Finally, the contribution was described by the matrix ${K}_{r}$ expressed into the segment reference frame, as follows:

Surgeon

with *P*_*ij*_ [6×6] submatrices defined as follows:

4 $$P_{iA} = \left[ {\begin{array}{*{20}c} 1 & 0 & 0 & 0 & b & { - a} \\ {} & 1 & 0 & { - b} & 0 & 0 \\ {} & {} & 1 & a & 0 & 0 \\ {} & {\left[ 0 \right]} & {} & 1 & 0 & 0 \\ {} & {} & {} & {} & 1 & 0 \\ {} & {} & {} & {} & {} & 1 \\ \end{array} } \right];P_{iB} \; = \;[0];\,P_{jA} \; = \;[0];\,P_{jB} = \left[ {\begin{array}{*{20}c} 1 & 0 & 0 & 0 & b & { - a} \\ {} & 1 & 0 & { - b} & 0 & 0 \\ {} & {} & 1 & a & 0 & 0 \\ {} & {\left[ 0 \right]} & {} & 1 & 0 & 0 \\ {} & {} & {} & {} & 1 & 0 \\ {} & {} & {} & {} & {} & 1 \\ \end{array} } \right]$$

and properties of submatrices such that ${K}_{rii}={K}_{rjj}$ and ${K}_{rij}={K}_{rji}$, as follows:

5 $$K_{rii} \; = \;\left[ {\begin{array}{*{20}c} {k_{11} } & 0 & 0 & 0 & {bk_{11} } & { - ak_{11} } \\ {} & {k_{22} } & 0 & { - bk_{22} } & 0 & {k_{26} } \\ {} & {} & {k_{22} } & {ak_{22} } & { - k_{26} } & 0 \\ {} & {} & {} & {\begin{array}{*{20}c} {k_{44} } \\ { + k_{22} \left( {a^{2} + b^{2} } \right)} \\ \end{array} } & { - ak_{26} } & { - bk_{26} } \\ {} & {sym} & {} & {} & {\begin{array}{*{20}c} {k_{55} } \\ { + b^{2} k_{11} } \\ \end{array} } & { - abk_{11} } \\ {} & {} & {} & {} & {} & {\begin{array}{*{20}c} {k_{55} } \\ { + a^{2} k_{11} } \\ \end{array} } \\ \end{array} } \right];\, K_{rij} \; = \;\left[ {\begin{array}{*{20}c} { - k_{11} } & 0 & 0 & 0 & { - bk_{11} } & {ak_{11} } \\ 0 & { - k_{22} } & 0 & {bk_{22} } & 0 & {k_{26} } \\ 0 & 0 & { - k_{22} } & { - ak_{22} } & { - k_{26} } & 0 \\ 0 & {bk_{22} } & { - ak_{22} } & {\begin{array}{*{20}c} { - k_{44} } \\ { - k_{22} \left( {a^{2} + b^{2} } \right)} \\ \end{array} } & { - ak_{26} } & { - bk_{26} } \\ { - bk_{11} } & 0 & {k_{26} } & {ak_{26} } & {\begin{array}{*{20}c} {k_{511} } \\ { - b^{2} k_{11} } \\ \end{array} } & {abk_{11} } \\ {ak_{11} } & { - k_{22} /2} & 0 & {bk_{22} /2} & {abk_{11} } & {\begin{array}{*{20}c} {k_{511} } \\ { - a^{2} k_{11} } \\ \end{array} } \\ \end{array} } \right]$$

The application concerned chrome-cobalt alloy rod of *ϕ* = 5.5 mm (Medtronic®) with Young modulus *e* of 2.45×10^11^ Pa and Poisson ratio *υ* of 0.3. Average geometrical properties of the construction were *a* = *b* = 30 mm for the offset, *L* = 35 mm for the rod span, and *α* = 5/6 for the shear coefficient of the cylindrical cross-section. Finally, numerical values of surgical instrumentation involving two rods, were as follows:

6 $$K_{rii} \; = \;10^{4} \left[ {\begin{array}{*{20}c} {3.3 \times 10^{4} } & 0 & 0 & 0 & {1 \times 10^{3} } & { - 1 \times 10^{3} } \\ {} & {580} & 0 & { - 17} & 0 & {10} \\ {} & {} & {580} & {17} & { - 10} & 0 \\ {} & {} & {} & {1.1} & { - 0.3} & { - 0.3} \\ {} & {sym} & {} & {} & {30} & { - 29} \\ {} & {} & {} & {} & {} & {30} \\ \end{array} } \right] \,K_{rij} \; = \;10^{4} \left[ {\begin{array}{*{20}c} { - 3 \times 10^{4} } & 0 & 0 & 0 & { - 996} & {996} \\ 0 & { - 580} & 0 & {17} & 0 & {10} \\ 0 & 0 & { - 580} & { - 17} & { - 10} & 0 \\ 0 & {17} & { - 17} & { - 1.1} & { - 0.3} & { - 0.3} \\ { - 996} & 0 & {10} & {0.3} & { - 26} & {30} \\ { - 996} & { - 290} & 0 & {8.7} & {30} & { - 26} \\ \end{array} } \right]$$

Boundary conditions between instrumented vertebral segments and free levels are implemented as scoliosis reduction coefficient, i.e., the capacity of the clinical practice to reduce the frontal deformation, which is translated in the software as an angle between the instrumented vertebra and the previous (LIV) or following (UIV) free level.

## Abbreviations

AIS: Adolescent idiopathic scoliosis
3D: Three-dimensional
SD: Standard deviation
CI: Confidence interval
IQR: Interquartile range
